# Critically ill patients with diabetes and Middle East respiratory syndrome: a multi-center observational study

**DOI:** 10.1186/s12879-021-05771-y

**Published:** 2021-01-19

**Authors:** Jesna Jose, Hasan M. Al-Dorzi, Awad Al-Omari, Yasser Mandourah, Fahad Al-Hameed, Musharaf Sadat, Eman Al Qasim, Basem Alraddadi, Abdulrahman Al Harthy, Ghaleb A. Al Mekhlafi, Abdullah Almotairi, Kasim Al Khatib, Ahmed Abdulmomen, Ismael Qushmaq, Anees A. Sindi, Ahmed Mady, Othman Solaiman, Rajaa Al-Raddadi, Khalid Maghrabi, Ahmed Ragab, Ayman Kharaba, Sarah Shalhoub, Abdulsalam M. Al-Aithan, Gajendra K. Vishwakarma, Atanu Bhattacharjee, Yaseen M. Arabi, The Saudi Critical Care Trials Group

**Affiliations:** 1 Department of Biostatistics and Bioinformatics, King Abdullah International Medical Research Center, Riyadh, Saudi Arabia; 2 Department of Mathematics & Computing, Indian Institute of Technology (ISM), Dhanbad, Jharkhand 826004 India; 3 Intensive Care Department, Ministry of National Guard Health Affairs, King Abdullah International Medical Research Center and King Saud Bin Abdulaziz University for Health Sciences, Riyadh, Saudi Arabia; 4 Department of Intensive Care, College of Medicine, Alfaisal University, Dr Sulaiman Al-Habib Group Hospitals, Riyadh, Saudi Arabia; 5 Military Medical Services, Ministry of Defense, Prince Sultan Military Medical City, Riyadh, Saudi Arabia; 6 Department of Intensive Care, College of Medicine, King Saud bin Abdulaziz University for Health Sciences, King Abdullah International Medical Research Center, King Abdulaziz Medical City, Jeddah, Saudi Arabia; 7 Department of Medicine, King Faisal Specialist Hospital and Research Center, Jeddah, Saudi Arabia; 8 Department of Medicine, University of Jeddah, Jeddah, Saudi Arabia; 9 Intensive Care Department, King Saud Medical City, Riyadh, Saudi Arabia; 10 Department of Intensive Care Services, Prince Sultan Military Medical City, Riyadh, Saudi Arabia; 11 Department of Critical Care Medicine, King Fahad Medical City, Riyadh, Saudi Arabia; 12 Intensive Care Department, Al-Noor Specialist Hospital, Makkah, Saudi Arabia; 13 Department of Critical Care Medicine, King Saud University, Riyadh, Saudi Arabia; 14 Section of Critical Care Medicine, Department of Medicine, King Faisal Specialist Hospital and Research Center, Jeddah, Saudi Arabia; 15 Department of Anesthesia and Critical Care, Faculty of Medicine, King Abdulaziz University, Jeddah, Saudi Arabia; 16 Tanta University Hospitals, Tanta, Egypt; 17 Intensive Care Department, King Faisal Specialist Hospital and Research Center, Riyadh, Saudi Arabia; 18 Department of Community Medicine, Faculty of Medicine, King Abdulaziz University, Jeddah, Saudi Arabia; 19 Intensive Care Department, King Fahd Hospital, Jeddah, Saudi Arabia; 20 Department of Critical Care, King Fahad Hospital, Ohoud Hospital, Al-Madinah, Saudi Arabia; 21 Department of Medicine, Division of Infectious Diseases, University of Western Ontario, London, Canada; 22 King Fahad Armed Forces Hospital, Jeddah, Saudi Arabia; 23 Department of Medicine, Critical Care Division, King Abdulaziz Hospital, Al Ahsa, Saudi Arabia; 24 Homi Bhaba National Institute, Section of Biostatistics, Centre for Cancer Epidemiology, Tata Memorial Centre, Navi Mumbai, India

**Keywords:** Acute respiratory distress syndrome, Coronavirus, Diabetes, Middle East respiratory syndrome

## Abstract

**Background:**

Diabetes is a risk factor for infection with coronaviruses. This study describes the demographic, clinical data, and outcomes of critically ill patients with diabetes and Middle East Respiratory Syndrome (MERS).

**Methods:**

This retrospective cohort study was conducted at 14 hospitals in Saudi Arabia (September 2012–January 2018). We compared the demographic characteristics, underlying medical conditions, presenting symptoms and signs, management and clinical course, and outcomes of critically ill patients with MERS who had diabetes compared to those with no diabetes. Multivariable logistic regression analysis was performed to determine if diabetes was an independent predictor of 90-day mortality.

**Results:**

Of the 350 critically ill patients with MERS, 171 (48.9%) had diabetes. Patients with diabetes were more likely to be older, and have comorbid conditions, compared to patients with no diabetes. They were more likely to present with respiratory failure requiring intubation, vasopressors, and corticosteroids. The median time to clearance of MERS-CoV RNA was similar (23 days (Q1, Q3: 17, 36) in patients with diabetes and 21.0 days (Q1, Q3: 10, 33) in patients with no diabetes). Mortality at 90 days was higher in patients with diabetes (78.9% versus 54.7%, *p* < 0.0001). Multivariable regression analysis showed that diabetes was an independent risk factor for 90-day mortality (odds ratio, 2.09; 95% confidence interval, 1.18–3.72).

**Conclusions:**

Half of the critically ill patients with MERS have diabetes; which is associated with more severe disease. Diabetes is an independent predictor of mortality among critically patients with MERS.

**Supplementary Information:**

The online version contains supplementary material available at 10.1186/s12879-021-05771-y.

## Background

The Middle East respiratory syndrome coronavirus (MERS-CoV) is a novel zoonotic virus that can lead to severe acute respiratory infection (SARI) and life-threatening multi-organ dysfunction. It was first isolated from a fatal case of pneumonia in Jeddah, Saudi Arabia in 2012 [1, 2]. Since then, community-acquired cases and clusters in healthcare settings have been reported mainly in Saudi Arabia [3, 4], but also other countries [5]. By the end of March 2020, the World Health Organization (WHO) reported 2553 confirmed cases in 27 countries (84.3% of cases in Saudi Arabia) with a case fatality rate of 34.4% [6]. MERS clinical presentation ranges from asymptomatic infection to rapidly progressive severe respiratory failure with multi-organ failure [7, 8]. Symptoms usually manifest after an incubation period of 2–14 days, with fever, cough, and dyspnea [7–9]. Admission to the intensive care unit (ICU) is frequently needed [7, 8].

Most severe MERS cases have been reported in older adults with chronic comorbidities, including diabetes mellitus [2, 7, 9–12]. One cohort study found that among 47 MERS patients, 68% had diabetes [7]. A case-control study demonstrated that diabetes was associated with an increased risk of MERS with an adjusted odds ratio [OR] of 6.99 (95% confidence interval [CI], 1.89–25.86) [11]. Diabetes has also been associated with increased mortality in MERS patients [9, 13]. In animal studies, diabetes was associated with a dysregulated immune response resulting in more severe and prolonged lung pathology following MERS-CoV infection [14].

Previous studies that evaluated diabetes in MERS had relatively small sample sizes, were mostly performed in single centers, and included a mix of critically and non-critically ill patients. We performed this study in a large cohort of critically ill patients with MERS, with the hypothesis that patients with diabetes and viral SARI would have a complicated course of illness and worse outcomes compared with patients with no diabetes. The objectives of this study are to describe the clinical presentation, management, and outcomes (including mortality and MERS-CoV RNA shedding) of the Middle East Respiratory Syndrome in critically ill patients with diabetes.

## Methods

We followed the STROBE (STrengthening the Reporting of OBservational studies in Epidemiology) guidelines in reporting this study.

### Patients and settings

This is a retrospective cohort study of adult (≥ 14-year-old) patients with SARI due to MERS-CoV who were admitted to the ICUs of 14 referral hospitals in Saudi Arabia between September 2012 and January 2018. The study was approved by the Institutional Review Boards of all participating centers. The characterization of this cohort has already been published earlier [15]. SARI was defined as an acute respiratory infection, with fever and cough onset within the preceding 10 days and clinical or radiologic lung involvement. The presence of MERS-CoV was detected by real-time reverse-transcriptase polymerase chain reaction assay (rRT-PCR) performed on nasopharyngeal swabs or sputum samples in non-intubated patients and tracheal aspirates or bronchoalveolar lavage in intubated patients as recommended by the Saudi Arabian Ministry of Health. Confirmatory laboratory testing required a positive PCR on at least two specific genomic targets (upE and ORF1a) or a single positive target (upE) with sequencing of a second target (RdRpSeq or NSeq). In patients with suspected MERS and negative rRT-PCR, testing was repeated at the discretion of the treating teams. For infection control purposes, follow-up respiratory samples were collected approximately 1–2 times per week in MERS-CoV positive patients [16] to assess clearance of MERS-CoV RNA [15].

### Data collection

Data were collected using the standardized International Severe Acute Respiratory and Emerging Infection Consortium (ISARIC) tool [17]. In this study, we included patients demographics, baseline characteristics, presenting symptoms, physiologic and laboratory parameters, and severity of illness on ICU admission assessed by the Sequential Organ Failure Assessment **(SOFA**) score [18]. We also described the management in the ICU, including the use of invasive and noninvasive ventilation, extracorporeal membrane oxygenation (ECMO), prone positioning, and selected medications.

The primary outcome was 90-day mortality. Other studied outcomes were mortality at 14 and 28 days and on ICU and hospital discharge and ICU and hospital length of stay (LOS). For patients who survived hospital discharge, the 90-day outcome was assessed by calling the patients. We also assessed the time to clearance of MERS-CoV RNA, which was defined as the time from the first performed rRT-PCR until the test was negative on two occasions, without a positive test afterward [15].

### Statistical analysis

In this study, patients were divided into two groups based on the history of preexisting diabetes mellitus, as reported by patients. We compared patients with diabetes to patients with no diabetes using the Student t-test or the Mann-Whitney U test for continuous variables based on normality assumption, and the chi-square test or Fisher’s exact test for categorical variables.

To examine the independent association of diabetes with 90-day mortality in MERS patients, we performed multivariable logistic regression analysis adjusting for certain variables selected based on their clinical relevance, excluding the ones which were in the exposure–causal pathway. The variables entered in the model were age, sex, asthma or chronic pulmonary disease, chronic neurological disease, immunosuppressant use before admission, body mass index (BMI), and SOFA score.

For the multivariable logistic regression analysis, 24% (84/350) of patients had missing data (BMI – 81/350, 23%, age – 2/350, 0.5%, and SOFA score – 3/350, 0.8%). Hence missing data were handled using the multiple imputation technique with 50 imputations. Two imputation methods were considered to support the imputation technique: (I) “Predictive mean matching” and (II) “Impute then Transform” approach. The data set had an arbitrary missing data pattern and it was assumed that the missing data were missing at random, such that the probability of a missing observation may depend on observed values but not on unobserved values. Predictive mean matching was used to impute missing values for these variables. For the imputation of BMI, we used the “impute then transform” approach instead of imputing BMI directly, such that we imputed the height and weight assuming the imputation model was oblivious of the relation between these two variables [19]. We reported the results of multivariable regression analysis without imputation (Model I: Complete case analysis) and with imputation (Model II: Multiple imputation).

Kaplan-Meier curves for the time to MERS-CoV RNA clearance were constructed censoring by hospital discharge or at 90 days whichever occurred first. The log-rank test was used to compare the median survival time between the groups. In addition, Kaplan-Meier curves for survival were also plotted and were censored at 90 days; the log-rank test was used to compare the time to survival between the groups. All statistical tests were two-sided with significance set at α < 0.05. All analyses were conducted using SAS version 9.4 (SAS Institute, Cary, NC).

## Results

### Characteristics of patients

During the study period, 350 patients with MERS SARI were admitted to the participating ICUs. Patients with diabetes constituted 48.9% of the cohort. Table 1 describes the characteristics and presenting symptoms of patients with diabetes and no diabetes. Compared to patients with no diabetes, those with diabetes were older (median age 61.0 years (Q1, Q3: 53.0, 72.0) versus median age 54.0 years (Q1, Q3: 35.0, 67.0), *p* < 0.0001) and more likely to have other comorbid conditions such as chronic renal disease (71 (41.5%) versus 39 (21.8%), *p* < 0.0001) and cardiac disease (95 (55.6%) versus 60 (40.5%), p < 0.0001). They were more likely to present with dyspnea and sputum production. The time from symptom onset to ICU admission was similar.

**Table 1:** Baseline characteristics of patients with diabetes and Middle East Respiratory Syndrome (MERS) compared to patients with no diabetes and MERS

Variables	Diabetes ***N*** = 171	No Diabetes ***N*** = 179	***P***-value
Age (year), median (Q1, Q3)	61.0 (53.0, 72.0)	54.0 (35.0, 67.0)	< 0.0001
BMI (Kg/m^2^), median (Q1, Q3)	29.3 (24.6, 33.3)	28.3 (24.1, 33.0)	0.31
Male sex – no. (%)	114 (66.7)	127 (70.9)	0.39
Healthcare worker – no. (%)	4 (2.3)	28 (15.6)	< 0.0001
Community-acquired – no. (%)	102 (59.6)	83 (46.4)	
Healthcare-associated (Non-healthcare worker) – no. (%)	65 (38.0)	68 (38.0)	
Days from the onset of symptoms to the emergency room, median (Q1, Q3)	5.0 (3.0, 8.0)	4.0 (2.0, 7.0)	0.30
Days from symptom onset to ICU admission, median (Q1, Q3)	7.0 (4.0, 10.5)	7.0 (4.0, 11.0)	0.66
Days from symptom onset to intubation, median (Q1, Q3)	8.0 (5.0, 12.0)	8.0 (5.0, 13.0)	0.37
**Presenting symptoms – no. (%)**			
**Lower respiratory**			
Dyspnea	136 (79.5)	123 (68.7)	0.02
Cough	122 (71.3)	117 (65.4)	0.23
With sputum	75 (43.9)	58 (32.4)	0.03
With bloody sputum	11 (6.4)	18 (10.1)	0.22
Chest pain	36 (21.1)	32 (17.9)	0.45
Wheezing	10 (5.8)	9 (5.0)	0.74
**Upper respiratory**			
Earache	2 (1.2)	1 (0.6)	0.62^
Rhinorrhea	6 (3.5)	11 (6.1)	0.25
Sore throat	23 (13.5)	24 (13.4)	0.99
**Systemic symptoms**			
Fever (temperature > 38 °C)	130 (76.0)	131 (73.2)	0.54
Myalgia or arthralgia	32 (18.7)	34 (19.0)	0.95
Headache	14 (8.2)	21 (11.7)	0.27
Fatigue	66 (38.6)	55 (30.7)	0.12
Abdominal pain	22 (12.9)	25 (14.0)	0.76
Lymphadenopathy	1 (0.6)	2 (1.1)	> 0.99^
Diarrhea	20 (11.7)	18 (10.1)	0.62
Vomiting/nausea	28 (16.4)	30 (16.8)	0.92
Altered consciousness/ confusion	44 (25.7)	30 (16.8)	0.04
Seizures	2 (1.2)	2 (1.1)	> 0.99^
**Other comorbidities – no. (%)**			
Chronic pulmonary disease (including asthma)	22 (12.9)	24 (13.4)	0.88
Liver disease	12 (7.0)	10 (5.6)	0.58
Chronic renal disease	71 (41.5)	39 (21.8)	< 0.0001
Chronic cardiac disease	95 (55.6)	43 (24.0)	< 0.0001
Chronic neurological disease	22 (12.9)	16 (8.9)	0.24
BMI > 30 (Kg/m^2^)	55 (45.5)	60 (40.5)	0.42
Rheumatologic disease	2 (1.2)	5 (2.8)	0.45^
Any malignancy including leukemia, lymphoma, or solid tumors	14 (8.2)	20 (11.2)	0.35
Immunosuppressant use before admission	6 (3.5)	15 (8.4)	0.06
SOFA score on ICU day 1, median (Q1, Q3)	9.0 (6.0, 12.0)	8.0 (5.0, 11.0)	0.02

*BMI* Body mass index, *ICU* intensive care unit, *Q1* first quartile, *Q3* third quartile, *SOFA* Sequential Organ Failure Assessment

The denominator of the percentage is the total number of subjects in the treatment group. For continuous variables, Mann-Whitney U test was used to calculate the P-value. For categorical variables, Chi-square test was used to calculate the *P*-value unless otherwise noted. ^Fisher’s exact test was used to calculate P-value

The laboratory findings are presented in Table 2. There were no differences in white blood cell and platelet counts between the two groups. Patients with diabetes had higher blood glucose (median 12.1 mmol/L (Q1,Q3: 9.9, 16.1) versus median 8.5 mmol/L (Q1,Q3: 6.5, 11.6), p < 0.0001), blood urea nitrogen (median 12.0 mmol/L (Q1,Q3: 7.3, 20.8) versus median 9.1 mmol/L (Q1,Q3: 4.1, 16.9), *p* = 0.0002) and creatinine (median 141.4 mmol/L (Q1,Q3: 91.0, 327.0) versus median 114.9 mmol/L (Q1,Q3: 67.0, 217.0), *p* = 0.0004).

**Table 2:** Physiological parameters on day 1 of admission to ICU in patients with diabetes and Middle East Respiratory Syndrome (MERS) compared to patients with no diabetes and MERS

Variables	Diabetes ***N*** = 179	No Diabetes ***N*** = 171	***P***-value
**Respiratory parameters on ICU day 1, median (Q1, Q3)**			
PaO_2_ (mmHg)	65.1 (56.0, 79.0)	71.0 (60.2, 86.4)	0.01
SaO_2_ (%)	92.0 (87.0, 95.0)	93.5 (90.0, 95.0)	0.004
FiO_2_,	0.7 (0.45, 1.0)	0.6 (0.45, 1.00)	0.24
PaO_2_/FiO_2_ ratio	98.0 (64.0, 160.0)	122.6 (73.4, 187.5)	0.02
**Extrapulmonary parameters on ICU day 1, median (Q1, Q3)**			
Mean arterial pressure (mmHg)	70.0 (61.0, 83.0)	70.0 (60.0, 80.0)	0.72
Leukocyte (× 10^9^/L)	7.90 (4.50, 11.60)	6.80 (4.20, 11.20)	0.31
Hemoglobin (g/dL)	10.4 (9.0, 12.50)	11.0 (8.5, 13.0)	0.48
Hematocrit	33.0 (28.55, 38.50)	35.0 (28.0, 40.0)	0.24
Platelets (×10^9^/L)	188.0 (117.0, 253.0)	168.5 (113.0, 253.0)	0.32
Glucose (mmol/L)	12.1 (9.9, 16.1)	8.5 (6.5, 11.6)	< 0.0001
Blood urea nitrogen (mmol/L)	12.0 (7.3, 20.8)	9.1 (4.1, 16.9)	0.0002
Creatinine (μmol/L)	141.4 (91.0, 327.0)	114.9 (67.0, 217.0)	0.0004
Bilirubin (μmol/L)	12.3 (7.8, 23.7)	12.0 (7.8, 22.9)	0.86

*PaO2* partial pressure of oxygen, *SaO2* Oxygen saturation, *FiO2* Fraction of inspired oxygen, *PaO2* FiO2 ratio, the ratio of the partial pressure of oxygen to the fraction of inspired oxygen, *ALT* alanine aminotransferase, *AST* aspartate transaminase, *Q1* first quartile, *Q3* third quartile

Data on variables were not available for some patients; the number of patients with missing data in the Diabetes group and the No diabetes group, respectively, were as follows: PaO2: 5 patients and 3 patients, SaO2: 3 patients and 3 patients, FiO2: 8 patients and 17 patients, PaO2: FiO2 ratio: 9 patients and 19 patients, MAP: 5 patients and 5 patients, Leukocyte: 5 patients and 9 patients, Hemoglobin: 5 patients and 10 patients, Hematocrit:7 patients, and 9 patients, Platelets: 6 patients and 5 patients, Glucose: 23 patients and 26 patients, Blood urea: 8 patients and 9 patients, Creatinine:4 patients and 2 patients, Bilirubin level: 22 patients and 23 patients

### Management in the ICU

Table 3 shows the management interventions performed during the ICU stay. More patients with diabetes were treated with non-invasive ventilation and with invasive mechanical ventilation (89.5% versus 81.6%, *p* = 0.04) than non-diabetics. The time from symptom onset to intubation was similar. In comparison to patients with no diabetes, there was more use of nitric oxide in patients with diabetes (28 (16.4%) versus 16 (8.9), p = 0.04, but less use of ECMO (6 (3.5%) versus 16 (8.9%), p = 0.04).

**Table 3:** Main interventions and outcomes in patients with diabetes and Middle East Respiratory Syndrome (MERS) compared to patients with no diabetes and MERS

Variables	Diabetes ***N*** = 171	No Diabetes ***N*** = 179	***P***-value
**Interventions**			
Non-invasive positive pressure ventilation – no. (%)	64 (37.4)	43 (24.0)	0.007
Invasive ventilation – no. (%)	153 (89.5)	146 (81.6)	0.04
Duration, median (Q1, Q3)	9.5 (4.0,17.0)	9.0 (4.0, 16.0)	0.68
Neuromuscular blockade – no. (%)	55 (32.2)	78 (43.6)	0.03
High-frequency oscillation ventilation – no. (%)	16 (9.4)	10 (5.6)	0.18
ECMO – no. (%)	6 (3.5)	16 (8.9)	0.04
Nitric oxide – no. (%)	28 (16.4)	16 (8.9)	0.04
Prone positioning – no. (%)	14 (8.2)	19 (10.6)	0.44
Duration, median (Q1, Q3)	3.0 (2.0,3.0)	3.0 (2.0, 3.0)	0.97
Any oxygen rescue therapy – no. (%)	73 (42.7)	84 (46.9)	0.43
Vasopressors – no. (%)	147 (86.0)	129 (72.1)	0.002
Duration, median (Q1, Q3)	6.5 (4.0,13.0)	6.0 (3.0, 14.0)	0.83
Blood transfusion – no. (%)	59 (34.5)	58 (32.4)	0.68
**Antivirals – no. (%)**			
Both interferon and ribavirin – no. (%)	70 (40.9)	47 (26.3)	0.02^
Interferon only – no. (%)	4 (2.3)	5 (2.8)	
Ribavirin only – no. (%)	10 (5.8)	8 (4.5)	
Oseltamivir – no. (%)	89 (52.0)	107 (59.8)	0.15
Corticosteroids – no. (%)	100 (58.5)	75 (41.9)	0.002
Renal replacement therapy – no. (%)	103 (60.2)	71 (39.7)	0.0001
Duration, median (Q1, Q3)	8.0 (4.0,14.0)	8.0 (3.0, 14.0)	0.92
Intravenous immunoglobins – no. (%)	9 (5.3)	15 (8.4)	0.25
Tracheostomy – no (%)	5 (2.9)	9 (5.0)	0.32
**Outcomes**			
ICU mortality – no. (%)	133 (77.8)	95 (53.1)	< 0.0001
Hospital mortality – no. (%)	136 (79.5)	102 (57.0)	< 0.0001
90-day mortality – no. (%)	135 (78.9)	98 (54.7)	< 0.0001
28-day mortality – no. (%)	127 (74.3)	90 (50.3)	< 0.0001
14-day mortality – no. (%)	89 (52.0)	76 (42.5)	0.07
ICU length of stay (days), median (Q1, Q3)	11.0 (6.0,19.0)	8.0 (5.0, 17.0)	0.05
Hospital length of stay (days), median (Q1, Q3)	18.0 (10.0,33.0)	20.0 (10.0, 37.0)	0.63

*ECMO* extracorporeal membrane oxygenation, *ICU* Intensive care unit. The denominator of the percentage is the total number of subjects in the group

For other organ support, more patients with diabetes had shock requiring vasopressors compared to those with no diabetes (86.0% versus 72.1%, *p* = 0.002). Corticosteroids were given more commonly in patients with diabetes (58.5% versus 41.9%, p = 0.002). Renal replacement therapy was provided more often in patients with diabetes (60.2% versus 39.7%, *p* = 0.0001). More patients with diabetes received ribavirin and interferon (40.9% versus 26.3%, *p* = 0.02).

### Outcomes of patients

The mortality at 90 days was higher in patients with diabetes (78.9% versus 54.7%, p < 0.0001) (Table 3**)**. On multivariable logistic regression analysis, in both the models (I and II) diabetes was associated with increased mortality (OR, 2.09; 95% CI, 1.18–3.72). Other predictors of mortality were age (OR per 1-year increment, 1.04; 95% CI, 1.02–1.06), female gender (OR 1.68; 95% CI, 1.06–2.67), SOFA (OR per 1-point increment 1.20; 95% CI, 1.14–1.26) (Table 4).

**Table 4:** Multivariable model to examine whether diabetes is an independent predictor of 90-day mortality in patients with Middle East Respiratory Syndrome (MERS). We adjusted for age, sex, asthma or chronic pulmonary disease, chronic neurological disease, immunosuppressant use before admission, body mass index (BMI), and SOFA score. Model I: Complete case analysis with no imputation (participants with any missing data are excluded). Model II: Multiple imputation (participants with missing data identified and replaced)

Variables	Model I (***N*** = 266)		Model II (***N*** = 350)	
	OR (95% CI)	***P***-value	OR (95% CI)	***P***-value
Diabetes	2.09 (1.18, 3.72)	0.005	2.13 (1.15, 3.95)	0.02
SOFA (per one-unit increase)	1.20 (1.14, 1.26)	< 0.0001	1.17 (1.12, 1.23)	< 0.0001
Female sex	1.68 (1.06, 2.67)	0.02	1.74 (1.09, 2.79)	0.02
Age (per one-year increase)	1.04 (1.02, 1.06)	0.0002	1.04 (1.02, 1.06)	< 0.0001
Chronic neurological disease	3.07 (0.80, 11.81)	0.07	3.40 (1.04, 11.14)	0.04
BMI (kg/m^2^) (per one-unit increase)	0.97 (0.90, 1.03)	0.23	0.96 (0.91, 1.02)	0.21
Immunosuppressant use before admission	1.24 (0.29, 5.26)	0.74	1.13 (0.35, 3.64)	0.84
Asthma or chronic pulmonary disease	1.20 (0.66, 2.20)	0.51	1.04 (0.57, 1.91)	0.90

*OR* Odds Ratio, *CI* Confidence Interval, *BMI* body mass index, *SOFA* Sequential Organ Failure Assessment

**Fig. 1:**
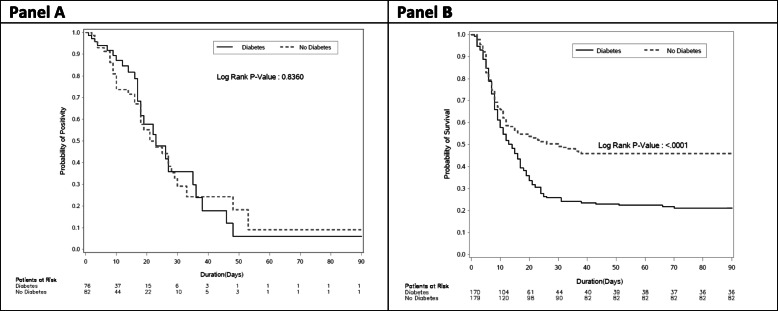
Panel **a**: Time-to-clearance of the Middle East respiratory syndrome coronavirus (MERS-CoV) RNA among patients with diabetes and no diabetes. Time to clearance was defined as the time taken from the date of ICU admission to having two negative RT-PCR tests not followed by positive test. Log-rank test is used to calculate the *P*-value. Panel **b:** Kaplan Meir curves for survival among patients with diabetes and no diabetes and Middle East respiratory syndrome (MERS)

ICU LOS was longer for patients with diabetes (Table 3**)**. The time to clearance of MERS-CoV RNA was similar in both groups (23 days (Q1, Q3: 17, 36) in patients with diabetes and 21.0 days (Q1, Q3: 10, 33) in patients with no diabetes). Figure 1 demonstrates the Kaplan Meir curves for time-to-clearance of MERS-CoV rRT-PCR and shows no difference between patients with diabetes and no diabetes.

## Discussion

Our study demonstrates that patients with diabetes constituted around half of the critically ill patients with MERS; MERS patients with diabetes presented with dyspnea and sputum production and were more likely to have respiratory failure requiring mechanical ventilation than those with no diabetes; and diabetes was an independent predictor of mortality in MERS. Viral shedding duration was similar in patients with diabetes and no diabetes.

Diabetes is a global health problem and leads to significant complications that increase the risk of morbidity and development of critical illness. Saudi Arabia is among the countries with high prevalence rates (> 30%) [20]. This may partly explain the high prevalence of diabetes in our cohort of critically ill MERS patients. In a Korean cohort of 186 patients with confirmed MERS patients, diabetes was present in 18.8% [9]. In our study, patients with diabetes presented with more severe respiratory symptoms and hypoxia, required mechanical ventilation more frequently, and were given nitric oxide as rescue therapy more often. They also required vasopressors more often. These patients were more likely to receive ribavirin and interferon (alpha or beta-1a) alone or in combination; these antivirals have not been associated with improved outcomes in MERS [21]. Recently, the MIRACLE trial demonstrated a reduction in mortality with the combination of lopinavir-ritonavir and interferon beta-1b; but none of patients in this cohort had received this combination [22].

Diabetes is associated with reduced neutrophil chemotaxis after stimulation [23] and blunted inflammatory response to endotoxemia [24]. These abnormalities are thought to be the reasons why diabetics have an increased risk of various infections [25]. For viral infections, diabetes has been associated with increased risk for hospitalization after H1N1 infection [26], ICU admission [26], and death [27]. Comorbidities, including diabetes, have been associated with increased mortality in MERS patients [28]. In a small cohort from two hospitals in Saudi Arabia, diabetes was present in 10.5% of survivors and 70.0% of non-survivors (*p* = 0.002) [29]. A study that evaluated MERS cases during the Korean outbreak found that diabetes was a risk factor for mortality on multivariate analysis (OR, 2.47; 95% CI, 1.06–5.72) [9]. Analysis of 1743 MERS cases found that patients with comorbidity (diabetes mellitus, cardiovascular disease, renal disease, or pulmonary disease) had higher mortality risk (adjusted hazard ratio, of 3.7; 95% CI, 2.6–5.7) [13]. In Severe Acute Respiratory Syndrome (SARS), diabetes (OR, 3.0; 95% CI, 1.4–6.3) and fasting blood glucose ≥7.0 mmol/L (OR, 3.3; 95%, CI 1.4–7.7) were independent predictors of death [30]. Studies on the association of diabetes with disease severity and outcome in COVID-19 have yielded mixed results [31–36]. In a meta-analysis, the risk of severe COVID-19 was not significantly increased in patients with diabetes (OR, 2.07; 95% CI, 0.89–4.82) [36]. Other studies found no association between diabetes and morality [32, 35].

It remains unclear how diabetes may contribute to increased disease severity and mortality in people infected with MERS-CoV. In a mouse model of MERS-CoV infection, diabetic mice had a prolonged phase of severe disease and delayed recovery compared to non-diabetic mice [14]. This was associated with delayed inflammation which lasted through 21 days after infection [14]. Diabetic mice exhibited fewer inflammatory monocyte/macrophages and CD4+ T cells and lower levels of TNF-a, IL-6, and IL-12b [14]. This may explain the findings of severe MERS in patients with diabetes.

Viral shedding was relatively prolonged in our MERS patients. However, the time to clearance of MERS-CoV RNA was similar in patients with diabetes and no diabetes. Prolonged shedding has been reported in MERS patients in other studies [37], and has been associated with corticosteroid use [38]. Corticosteroids were used more commonly in patients with diabetes in the current study.

The findings of this study should be interpreted in light of its strengths and weaknesses. The strengths include that it is the largest cohort of critically ill patients with MERS. The limitations are related to the nature of the database and include diabetes diagnosis by history and absence of data on glycemic measures, such as type of diabetes, hemoglobin A1c, glucose control during hospitalization, and prior or current diabetes medications. In addition, glucose levels in patients with no diabetes were elevated, suggesting that some patients had stress hyperglycemia or undiagnosed diabetes. This may affect the associations between diabetes and various outcomes. Given the high prevalence of diabetes in Saudi Arabia, the results of our study may not be generalizable to populations of lower diabetes prevalence.

## Conclusion

Diabetes was highly prevalent in a cohort of critically ill patients with MERS. Patients with diabetes had more severe illness. Diabetes was an independent predictor of mortality.

### Ethics approval and consent to participants

The study was approved by the Ministry of National Guard Health Affairs Institutional Review Board (IRB- RC14–025-R) and by the IRBs of all participating sites (Table S2: Supplement). Informed consent was waived by the IRB because of the retrospective and observational nature of the study.

## Supplementary Information

**Additional file 1.**

## Data Availability

The dataset(s) supporting the conclusions of this article will be made available on the approval of the PI and according to the rules of King Abdullah International Medical Research Center (KAIMRC).

## References

[1] Zaki AM, van Boheemen S, Bestebroer TM, Osterhaus AD, Fouchier RA (2012). Isolation of a novel coronavirus from a man with pneumonia in Saudi Arabia. N Engl J Med.

[2] Arabi YM, Balkhy HH, Hayden FG, Bouchama A, Luke T, Baillie JK, Al-Omari A, Hajeer AH, Senga M, Denison MR (2017). Middle East respiratory syndrome. N Engl J Med.

[3] Assiri A, McGeer A, Perl TM, Price CS, Al Rabeeah AA, Cummings DA, Alabdullatif ZN, Assad M, Almulhim A, Makhdoom H (2013). Hospital outbreak of Middle East respiratory syndrome coronavirus. N Engl J Med.

[4] Oboho IK, Tomczyk SM, Al-Asmari AM, Banjar AA, Al-Mugti H, Aloraini MS, Alkhaldi KZ, Almohammadi EL, Alraddadi BM, Gerber SI (2015). 2014 MERS-CoV outbreak in Jeddah--a link to health care facilities. N Engl J Med.

[5] Cho S, Kang J, Ha Y, Park G, Lee J, Ko J, Lee J, Kim J, Kang C, Jo I (2016). MERS-CoV outbreak following a single patient exposure in an emergency room in South Korea: an epidemiological outbreak study. Lancet (London, England).

[6] Cai Y, Yu SQ, Postnikova EN, Mazur S, Bernbaum JG, Burk R, Zhang T, Radoshitzky SR, Muller MA, Jordan I (2014). CD26/DPP4 cell-surface expression in bat cells correlates with bat cell susceptibility to Middle East respiratory syndrome coronavirus (MERS-CoV) infection and evolution of persistent infection. PLoS One.

[7] Assiri A, Al-Tawfiq J, Al-Rabeeah A, Al-Rabiah F, Al-Hajjar S, Al-Barrak A, Flemban H, Al-Nassir W, Balkhy H, Al-Hakeem R (2013). Epidemiological, demographic, and clinical characteristics of 47 cases of Middle East respiratory syndrome coronavirus disease from Saudi Arabia: a descriptive study. Lancet Infect Dis.

[8] Saad M, Omrani A, Baig K, Bahloul A, Elzein F, Matin M, Selim M, Al Mutairi M, Al Nakhli D, Al Aidaroos A (2014). Clinical aspects and outcomes of 70 patients with Middle East respiratory syndrome coronavirus infection: a single-center experience in Saudi Arabia. Int J Infect Dis.

[9] Choi WS, Kang C-I, Kim Y, Choi J-P, Joh JS, Shin H-S, Kim G, Peck KR, Chung DR, Kim HO (2016). Clinical presentation and outcomes of Middle East respiratory syndrome in the Republic of Korea. Infect Chemother.

[10] Al-Tawfiq JA, Hinedi K, Ghandour J, Khairalla H, Musleh S, Ujayli A, Memish ZA (2014). Middle East respiratory syndrome coronavirus: a case-control study of hospitalized patients. Clin Infect Dis.

[11] Alraddadi BM, Watson JT, Almarashi A, Abedi GR, Turkistani A, Sadran M, Housa A, Almazroa MA, Alraihan N, Banjar A (2016). Risk factors for primary Middle East respiratory syndrome coronavirus illness in humans, Saudi Arabia, 2014. Emerg Infect Dis.

[12] Alqahtani F, Aleanizy F, Mohamed RAEH, Alanazi M, Mohamed N, Alrasheed M, Abanmy N, Alhawassi T. Prevalence of comorbidities in cases of Middle East respiratory syndrome coronavirus: a retrospective study. Epidemiol Infect. 2019;147:1–5. 10.1017/S0950268818002923 PMC6518603 30394248

[13] Yang Y-M, Hsu C-Y, Lai C-C, Yen M-F, Wikramaratna PS, Chen H-H, Wang T-H. Impact of comorbidity on fatality rate of patients with Middle East respiratory syndrome. Sci Rep. 2017;7:11307. 10.1038/s41598-017-10402-1 PMC5596001 28900101

[14] Kulcsar KA, Coleman CM, Beck SE, Frieman MB. Comorbid diabetes results in immune dysregulation and enhanced disease severity following MERS-CoV infection. JCI insight. 2019;4(20). 10.1172/jci.insight.131774 PMC6824443 31550243

[15] Arabi YM, Al-Omari A, Mandourah Y, Al-Hameed F, Sindi AA, Alraddadi B, Shalhoub S, Almotairi A, Al Khatib K, Abdulmomen A (2017). Critically ill patients with the Middle East respiratory syndrome: a multicenter retrospective cohort study. Crit Care Med.

[16] Mawatari H, Yoneda M, Fujita K, Nozaki Y, Shinohara Y, Sasaki H, Iida H, Takahashi H, Inamori M, Abe Y (2010). Association between phospholipids and free cholesterol in high-density lipoprotein and the response to hepatitis C treatment in Japanese with genotype 1b. J Viral Hepat.

[17] Chowell G, Blumberg S, Simonsen L, Miller MA, Viboud C (2014). Synthesizing data and models for the spread of MERS-CoV, 2013: key role of index cases and hospital transmission. Epidemics.

[18] Vincent J, Moreno R, Takala J, Willatts S, De Mendonça A, Bruining H, Reinhart C, Suter P, Thijs L (1996). The SOFA (Sepsis-related Organ Failure Assessment) score to describe organ dysfunction/failure. On behalf of the Working Group on Sepsis-Related Problems of the European Society of Intensive Care Medicine. Intensive Care Med.

[19] Von Hippel PT (2009). How to impute interactions, squares and ther transformed variables. Sociol Methodol.

[20] Meo S, Usmani A, Qalbani E (2017). Prevalence of type 2 diabetes in the Arab world: impact of GDP and energy consumption. Eur Rev Med Pharmacol Sci.

[21] Arabi Y, Shalhoub S, Mandourah Y, Al-Hameed F, Al-Omari A, Al Qasim E, Jose J, Alraddadi B, Almotairi A, Al Khatib K. Ribavirin and interferon therapy for critically ill patients with Middle East respiratory syndrome: a multicenter observational study. Clin Infect Dis. 2019. 10.1093/cid/ciz544 PMC7108209 31925415

[22] Arabi YM, Asiri AY, Assiri AM, Balkhy HH, Al Bshabshe A, Al Jeraisy M, Mandourah Y, Azzam MHA, Bin Eshaq AM, Al Johani S (2020). Interferon Beta-1b and Lopinavir-ritonavir for Middle East respiratory syndrome. N Engl J Med.

[23] Delamaire M, Maugendre D, Moreno M, Le Goff MC, Allannic H, Genetet B (1997). Impaired leucocyte functions in diabetic patients. Diabet Med.

[24] Andreasen AS, Pedersen-Skovsgaard T, Berg RM, Svendsen KD, Feldt-Rasmussen B, Pedersen BK, Moller K (2010). Type 2 diabetes mellitus is associated with impaired cytokine response and adhesion molecule expression in human endotoxemia. Intensive Care Med.

[25] Muller LM, Gorter KJ, Hak E, Goudzwaard WL, Schellevis FG, Hoepelman AI, Rutten GE (2005). Increased risk of common infections in patients with type 1 and type 2 diabetes mellitus. Clin Infect Dis.

[26] Allard R, Leclerc P, Tremblay C, Tannenbaum TN (2010). Diabetes and the severity of pandemic influenza a (H1N1) infection. Diabetes Care.

[27] Hanslik T, Boelle PY, Flahault A (2010). Preliminary estimation of risk factors for admission to intensive care units and for death in patients infected with a(H1N1)2009 influenza virus, France, 2009-2010. PLoS Curr.

[28] Park J-E, Jung S, Kim A, Park J-E. MERS transmission and risk factors: a systematic review. BMC Public Health. 2018;18. 10.1186/s12889-018-5484-8 PMC5930778 29716568

[29] Sherbini N, Iskandrani A, Kharaba A, Khalid G, Abduljawad M, Al-Jahdali H (2017). Middle East respiratory syndrome coronavirus in Al-Madinah City, Saudi Arabia: demographic, clinical and survival data. J Epidemiol Global Health.

[30] Yang J, Feng Y, Yuan M, Yuan S, Fu H, Wu B, Sun G, Yang G, Zhang X, Wang L (2006). Plasma glucose levels and diabetes are independent predictors for mortality and morbidity in patients with SARS. Diabetic Med.

[31] Zhang J, Dong X, Cao Y, Yuan Y, Yang Y, Yan Y, Akdis C, Gao Y. Clinical characteristics of 140 patients infected by SARS-CoV-2 in Wuhan, China. Allergy. 2020. 10.1111/all.14238 32077115

[32] Zhou F, Yu T, Du R, Fan G, Liu Y, Liu Z, Xiang J, Wang Y, Song B, Gu X. Clinical course and risk factors for mortality of adult inpatients with COVID-19 in Wuhan, China: a retrospective cohort study. Lancet (London, England). 2020. 10.1016/S0140-6736(20)30566-3 PMC7270627 32171076

[33] Guan W, Ni Z, Hu Y, Liang W, Ou C, He J, Liu L, Shan H, Lei C, Hui D. Clinical characteristics of coronavirus disease 2019 in China. N Engl J Med. 2020. 10.1056/NEJMoa2002032 PMC7092819 32109013

[34] Wu Z, McGoogan J. Characteristics of and important lessons from the coronavirus disease 2019 (COVID-19) outbreak in China: summary of a report of 72 314 cases from the Chinese Center for Disease Control and Prevention. JAMA. 2020. 10.1001/jama.2020.2648 32091533

[35] Wu C, Chen X, Cai Y, Xia Ja, Zhou X, Xu S, Huang H, Zhang L, Zhou X, Du C. Risk Factors Associated With Acute Respiratory Distress Syndrome and Death in Patients With Coronavirus Disease 2019 Pneumonia in Wuhan, China. JAMA Internal Med. 180(7):934–43. 10.1001/jamainternmed.2020.0994 PMC7070509 32167524

[36] Yang J, Zheng Y, Gou X, Pu K, Chen Z, Guo Q, Ji R, Wang H, Wang Y, Zhou Y. Prevalence of comorbidities in the novel Wuhan coronavirus (COVID-19) infection: a systematic review and meta-analysis. Int J Infect Dis. 2020. 10.1016/j.ijid.2020.03.017 PMC7194638 32173574

[37] Bin S, Heo J, Song M, Lee J, Kim E, Park S, Kwon H, Kim S, Kim Y, Si Y (2016). Environmental contamination and viral shedding in MERS patients during MERS-CoV outbreak in South Korea. Clin Infect Dis.

[38] Arabi Y, Mandourah Y, Al-Hameed F, Sindi A, Almekhlafi G, Hussein M, Jose J, Pinto R, Al-Omari A, Kharaba A (2018). Corticosteroid therapy for critically ill patients with Middle East respiratory syndrome. Am J Respir Crit Care Med.

