# Supplementary material for: Critically ill patients with diabetes and Middle East respiratory syndrome: a multi-center observational study
Source: BMC Infect Dis. 2021 Jan 19;21:84. doi: 10.1186/s12879-021-05771-y (PMC7814976; doi:10.1186/s12879-021-05771-y)
Supplement: Supplementary file 1 — Additional file 1. [file 12879_2021_5771_MOESM1_ESM.docx]

**Supplementary appendix to**

**Critically ill patients with diabetes and Middle East respiratory syndrome: A multi-center observational study**

**Table S1 Collaborators**

| **Center** | **Names** |
| --- | --- |
| Saudi Arabia-The Saudi Critical Care Trials group |  |
| King Saud bin Abdulaziz University for Health Sciences and King Abdullah International Medical Research Center, Riyadh | Yaseen Arabi  Abdulaziz Aldawood  Hanan Balkhy  Mashael Al Ahmadi  Eman Al Qasim  Jesna Jose |
| King Fahad Medical City, Riyadh | Abdullah Al Motairi |
| Prince Sultan Cardiac Center, Riyadh | Ghaleb A. Almekhlafi  Yasser Mandourah  Sahar Hassan  Abid Alwan  Raylin Cabal  Rima E Mahamed  Khaloud M. Harbi  Abdulelah Ala Haidary  Shatha Moayad Awad |
| King Saud Medical City, Riyadh | Abelrahman Al-Harthy  Ahmed Fouad Mady   Omar Elsayed Ramadan  Muhammed Asim Rana  Basim Raafat Huwait  Mohamed Ali Al-Odat  Waleed Tharwat Al-Atreeby |
| King Faisal Specialist Hospital and Research Centre, Riyadh | Othman Solaiman |
| King Saud University, Riyadh | Ahmed Abdulmomen  Muhammed Fares  Mazen Barry |
| Security Forces Hospital, Riyadh | Awad Al Omari |
| King Abdulaziz Medical City, Jeddah | Fahad Al-Hameed  Jalal Al Refai |
| King Fahd Armed Forced Hospital, Jeddah | Sarah Shalhoub |
| King Faisal Specialist Hospital and Research Centre, Jeddah | Basem M Alraddadi  Rashed E Alrehaili  Sarah Batawi |
| King Fahad Hospital Group, Jeddah | Anees Sindi  Rajaa Al-Raddadi  Ahmed Rajab  Omimah Shabouni  Abeer Mustafa Housa  Amal AbdulallaTurkistani  Abdullatif Ayesh Almarashi  Amaal Ali Sarraj  Salwa Awad Own  Sara Mohammed AlJeaid  Wijdan Abdulalkareem Baeshen |
| AlNoor Specialist Hospital, Makkah | Kasim Al Khatib  Hamdy Badr  Majduldeen Azzo |
| King Abdulaziz Medical City-Alahsa | Abdulsalam Alaithan |
| King Fahad Hospital, Madinah | Ayman Kharaba  Noah Noor |
| **United Kingdom** |  |
| Infectious Diseases Data Observatory, Oxford University | Laura Merson |
| Canada |  |
| Sunnybrook Health Sciences Centre, Canada | Robert Fowler |
| United States of America |  |
| University of Virginia School of Medicine, USA | Frederick Hayden |

**Table S2: List of ethics committees of all participating sites**

| **Site Name** | **Ethics Committee** | **Reference No** |
| --- | --- | --- |
| King Saud bin Abdulaziz University for Health Sciences and King Abdullah International Medical Research Center, | Institutional Review Board, National Guard Health Affairs | RC14-025-R |
| King Abdulaziz Medical City, Riyadh |  |  |
| King Abdulaziz Hospital, Ahsa |  |  |
| King Abdulaziz Medical City, Jeddah |  |  |
| Security Forces Hospital, Riyadh | Research Committee, Security Forces Hospital Program | RS-AA/15 |
| King Saud Medical City, Riyadh | Research centre, King Saud Medical city | 2141666-1437 |
| King Saud University, Riyadh | Institutional Review Board, King Saud University-College of Medicine | 14-4301-IRB |
| King Fahad Hospital Group, Jeddah | Ethics Committee, Ministry of Health | A-00258 |
| King Fahad Hospital, Madinah |  |  |
| AlNoor Specialist Hospital, Makkah | Ethics Committee, Ministry of Health | 53429 |
| King Fahad Medical City, Riyadh | Institutional Review Board, King Fahad Medical City | H-01-R-012 |
| King Faisal Specialist Hospital & Research Centre, Jeddah | Research Ethics Committee, Office of Research Affairs | RC-J/182/36 |
| King Faisal Specialist Hospital & Research Centre, Riyadh |  |  |
| Prince Sultan Military Medical City, Riyadh | Research Ethics Committee | HAP-01-R-015 |
| King Fahd Armed Forced Hospital, Jeddah |  |  |
